# Carcinogenicity of ethylene oxide and 1,2-propylene oxide upon intragastric administration to rats.

**DOI:** 10.1038/bjc.1982.303

**Published:** 1982-12

**Authors:** H. Dunkelberg

## Abstract

**Images:**


*Br. J. Cancer* (1982) **46**, 924

### CARCINOGENICITY OF ETHYLENE OXIDE AND 1,2-PROPYLENE OXIDE UPON INTRAGASTRIC ADMINISTRATION TO RATS

H. DUNKELBERG

*From the Institute of Hygiene, University of Mainz, Hochhaus am Augustusplatz, D-6500 Mainz, Germany*

Received 24 March 1982 Accepted 24 August 1982

**Summary.—Ethylene oxide and 1,2-propylene oxide were each administered intragastrically by gavage at 2 dosages (30 and 7·5 mg/kg body wt; 60 and 15 mg/kg body wt respectively) to groups of 50 female Sprague–Dawley rats twice weekly for a period of nearly 3 years using salad oil as the solvent. Both compounds induced local tumours, mainly squamous-cell carcinomas of the forestomach, dependent on the dosage. The first tumour occurred in the 79th week both in the group treated with ethylene oxide and in that treated with 1,2-propylene oxide. The following tumour rates resulted: ethylene oxide 62 and 16%; 1,2-propylene oxide 40 and 4%. In addition carcinomata *in situ*, papillomas and reactive changes of the squamous epithelium of the forestomach were observed in other animals, but neither ethylene oxide nor 1,2-propylene oxide induced tumours at sites away from the point of administration.**

THE PRESENT ANNUAL CONSUMPTION of ethylene oxide in Western Europe stands at around 2·5 million tonnes (Schönfeldt, 1976), of which most is used in the production of various organic compounds. Ethylene oxide is also used for the fumigation of food and sterilization in the field of medicine. The carcinogenic risk is of particular importance in man. Indications of possible carcinogenic activity of ethylene oxide arise primarily from the mutagenic efficiency which has been proven by various methods of testing (see review by Glaser, 1979; Wolman, 1979; Ehrenberg & Hussain, 1981). Teratogenic effects could also be induced by ethylene oxide (La Borde & Kimmel, 1981). The alkylation of DNA and RNA or the mononucleotides by ethylene oxide and 1,2-propylene oxide has been investigated by Lawley & Wallick (1957), Fraenkel-Conrat (1961), Windmueller & Kaplan (1961), Lawley & Jarman (1972) and Ehrenberg *et al.* (1974). Further evidence of carcinogenic activity of ethylene oxide was provided by Reyniers *et al.* (1964) who observed, in an uncontrolled study, a higher incidence of tumours in female mice after the unintentional use of bedding which had been sterilized by ethylene oxide. Hogstedt *et al.* (1978, 1979*a, b*) reported an incidence of tumours in people whose occupation involved exposure to ethylene oxide. Carcinogenic activity of 1,2-propylene oxide upon s.c. administration to rats (Walpole, 1958) and mice (Dunkelberg, 1979, 1981) has been demonstrated, and ethylene oxide also showed a weak carcinogenic effect after s.c. application (Dunkelberg, 1979, 1981).

The aim of the experiment described here was to test ethylene oxide and 1,2-propylene oxide for carcinogenic activity by intragastric administration to rats.

### MATERIALS AND METHODS

*Purity of test substances.*—According to the producer's data (J.T. Baker Chemicals BV, Deventer, Netherlands) the ethylene oxide was 99·7% pure and the 1,2-propylene oxide (Merck-Schuchardt AG, München, Germany) 99% pure. The test substances as well as the solvent Livio oil (Union Deutscher Lebensmittelwerke, Hamburg, Germany) were first tested for impurities of polycyclic aromatic hydrocarbons according to a procedure described by Druckrey *et al.* (1966). There was no evidence of such impurity. We recorded in addition the infra-red spectra of the test substances and examined them for agreement with the reference spectra. Discrepancies which indicated impurities could not be established. The purity of the substances was additionally checked by gas chromatography.

*Experimental groups*

The experiment was performed on specifically pathogen-free female Sprague–Dawley rats (Ivanovas, Kisslegg, Germany), which were about 100 days old at the beginning of the experiment. They were confined in Macrolon cages type III with a cover of stainless steel wire. Sawdust was used as bedding. The rats were fed with pellets of the Altromin standard diet no. 1313 (Altromin GmbH, Lage, Germany) and tap water contained in Macrolon bottles.

The test substances, ethylene oxide and 1,2-propylene oxide, were each administered to groups of 50 rats per dosage. Two dosages of both compounds were tested. A group of 50 rats was treated with the solvent oil alone and another was left untreated. As a positive control a group was treated with β-propiolactone.

The animals were confined in air-conditioned rooms, and the group treated with β-propiolactone was situated in a room separate from the other groups. The substances were administered intragastrically twice weekly to rats with empty stomachs. Accordingly the food was removed from the cages about 16–18 h before the treatment. The test substances were dissolved in oil immediately before the treatment, so that the single dose according to the average weight was contained in 1 ml (Table I). Because of the low boiling-point of ethylene oxide, 1,2-propylene oxide and β-propiolactone the solutions were kept in cooled containers. β-Propiolactone, scarcely soluble in oil, was administered as a suspension. The test substances were stored and the solutions prepared in a laboratory away from the cages. The administration took place with sterile disposable syringes with stomach tubes of 1·5 mm × 80 mm. The animals were treated and observed for their lifespan and were examined thoroughly at necropsy. Pathologically noteworthy organs were removed and fixed in 5% buffered formalin. The stomach and the adjoining part of duodenum were opened along the greater curvature and, if change in the epithelium could be observed, pinned flat on a plate. After fixation these preparations were cut into 6 longitudinal strips and embedded. Paraplast sections were stained with haematoxylin and eosin. The diagnoses were confirmed histologically. Between the 79th and 82nd week several rats in the various groups contracted pneumonia, during which time the administrations were interrupted. We treated all rats s.c. with 2 × 100 mg chloramphenicol and subsequently added tylosin-tartrate (Eli Lilly GmbH, Germany) in a concentration of 0·5 g/l to the drinking water over a period of 3 weeks. The experiment lasted 150 weeks during which time the ethylene oxide groups received the test substances 214 times, the 1,2-propylene oxide groups 219 times and the β-propiolactone group 100 times. The average total dosage in mg/kg body wt for the various groups is recorded in Table I.

### RESULTS

The survival rates of the rats treated with ethylene oxide and 1,2-propylene oxide are comparable to those of the control groups with the exception of those treated with the higher dose of ethylene oxide. The latter died earlier from tumours. The survival rate of rats treated with β-propiolactone fell distinctly as tumours started to develop (see Figs 1 & 2).

The first animal to develop a squamous-cell carcinoma of the forestomach (Fig. 3) was observed after 32 weeks of the experiment in the β-propiolactone group. Between the 52nd and 84th week the majority of the animals in this group died of a carcinoma of the stomach. In total the 100 intragastric administrations of β-propiolactone led to stomach tumours in 46/50 rats. In most of the cases mesenteric metastases and metastases to the diaphragm as well as infiltration into the liver were observed. Animals with stomach

TABLE I.—*Experimental groups for carcinogenicity testing of ethylene oxide and* 1,2-*propylene oxide by intragastric administration to Sprague–Dawley rats*

| Group | Single dose (mg/kg body wt) | Average total dose (mg/kg body wt) | No. of animals |
|---|---|---|---|
| Ethylene oxide I | 30·0 | 5112 | 50 |
| Ethylene oxide II | 7·5 | 1186 | 50 |
| 1,2-Propylene oxide I | 60·0 | 10798 | 50 |
| 1,2-Propylene oxide II | 15·0 | 2714 | 50 |
| Oil (vehicle) | 1·0 ml | — | 50 |
| Untreated | — | — | 50 |
| β-Propiolactone | 30·0 | 2868 | 50 |

FIG. 1.—Length of survival of the rats treated with ethylene oxide.

tumours were also observed in the groups which were administered ethylene oxide and 1,2-propylene oxide but not in those which were administered only the oil or left untreated. (Incidences and histological findings of the tumours are illustrated in Figs 7 and 8). In most cases the stomach tumours were squamous-cell carcinomas of the forestomach. However, we observed tumours of the glandular stomach in animals treated with either compound. One of these (a fibrosarcoma) occurred in a rat treated with the higher dose of ethylene oxide and it infiltrated the liver and mesentery. In another animal a pyloric adenocarcinoma was present in addition to a squamous-cell carcinoma. In the 1,2-propylene oxide I group one animal had an adenocarcinoma of the pylorus. The first tumour was observed in the 79th week in both the ethylene oxide I and in the 1,2-propylene oxide I groups. We also observed carcinomata *in situ* (Fig. 4), early carcinomas (Fig. 5) and reactive changes of the squamous epithelium of the stomach such as hyperkeratosis, hyperplasia and papillomas in some of the rats which were administered ethylene oxide or 1,2-propylene oxide and which died primarily of another cause. (The corresponding frequencies can be seen from Figs 7 and 8. In the Figs each animal is classified once only; if carcinoma and other findings (*e.g.*

FIG. 2.—Length of survival of the rats treated with 1,2-propylene oxide.

FIG. 3.—Squamous-cell carcinoma of the forestomach showing formation of horny pearls and invasive growth in the glandular stomach (rat from the group treated with β-propiolactone). H. & E. ×55.

FIG. 4.—Carcinoma *in situ* of the forestomach in a rat from the ethylene oxide I group. H. & E. ×200.

FIG. 5.—Early squamous-cell carcinoma of the forestomach (ethylene oxide I group) with onset of infiltrating growth. H. & E. ×100.

FIG. 6.—Metastasis to a regional lymph node in a rat from the ethylene oxide I group with a squamous-cell carcinoma of the forestomach. H. & E. ×200.

papilloma) occurred simultaneously in the same animal, it was classified only under the carcinoma group.)

As far as could be ascertained the stomach tumours induced by β-propiolactone were the cause of death but tumours in the mammary gland, pituitary and uterus were the cause of death in some of the rats treated with ethylene oxide and 1,2-propylene oxide (Table II). In other rats the stomach tumours were the cause of death. These rats comprised 15 animals from the ethylene oxide I group which had large stomach tumours, of which 10 revealed metastases (Fig. 6) and invasive growth into neighbouring organs; and 4 animals treated with 1,2-propylene oxide I which had 4 large tumours of which 2 showed infiltrative growth.

It can be seen from Figs 7 and 8 that ethylene oxide and 1,2-propylene oxide lead to the induction of the same type of tumours. Despite a lower dosage (*cf.* Table I) the number of animals with carcinomas of the stomach in the ethylene oxide I group is clearly greater than in the 1,2-propylene oxide I group, and the rate of reactive changes in the squamous epithelium of the stomach in the 1,2-propylene oxide I group is correspondingly greater.

The frequencies of tumours away from the route of administration in the groups treated with ethylene oxide and 1,2-propylene oxide and the control groups can be seen in Table II. With regard to any type of tumours occurring away from the route of application no clear increase could be observed in the groups treated with ethylene oxide and 1,2-propylene oxide in comparison with controls.

### DISCUSSION

Our investigations clearly show that the administration of ethylene oxide and 1,2-propylene oxide by intragastric adminis-

TABLE II.—*Incidence of tumours occurring distant from the route of administration*

| Group | Site | Tumour (No. of animals) |
|---|---|---|
| Ethylene oxide I | Mammary gland | Adenofibroma (1) |
| | | Fibroadenoma (8) |
| | | Fibroma (1) |
| | | Adenocarcinoma (2) |
| | | Sarcoma (2) |
| | Uterus | Mesenchymoma (1) |
| | | Leiomyoma (1) |
| | | Leiomyofibrosarcoma (3) |
| | | Malignant mesenchymoma (1) |
| | | Sarcoma (1) |
| | | Squamous-cell carcinoma (2) |
| | Intestine | Mesothelioma (1) |
| | Pituitary | Adenoma (1) |
| | Ovary | Granulosa-theca cell tumour (1) |
| | S.c. tissue | Mesenchymal tumour (1) |
| | Lymphatic tissue | Malignant lymphoma (1) |
| | Nervous system | Malignant schwannoma (1) |
| Ethylene oxide II | Mammary gland | Adenofibroma (13) |
| | | Fibroadenoma (4) |
| | | Fibroma (1) |
| | | Adenocarcinoma (3) |
| | Uterus | Malignant mesenchymoma (1) |
| | | Leiomyofibrosarcoma (2) |
| | Ovary | Malignant granulosa-theca-cell tumour (1) |
| | Lymphatic tissue | Malignant lymphoma (3) |
| 1,2-Propylene oxide I | Mammary gland | Adenofibroma (6) |
| | | Fibroadenoma (7) |
| | | Fibroma (2) |
| | | Adenocarcinoma (3) |
| | | Haemangioendothelioma (1) |
| | Uterus | Leiomyofibrosarcoma (1) |
| | Ovary | Granulosa-theca-cell tumour (1) |
| | Vagina | Squamous-cell carcinoma (1) |
| | Pituitary | Adenoma (3) |
| | Adrenal gland | Phaeochromocytoma (1) |
| 1,2-Propylene oxide II | Mammary gland | Adenofibroma (7) |
| | | Fibroadenoma (3) |
| | | Fibroma (6) |
| | | Adenocarcinoma (2) |
| | Uterus | Adenocarcinoma (1) |
| | | Leiomyosarcoma (1) |
| | | Leiomyofibrosarcoma (1) |
| | Intestine | Adenocarcinoma (1) |
| | | Adenosarcoma (1) |
| | Adrenal gland | Cortical adenocarcinoma (1) |
| | Lymphatic tissue | Malignant Lymphoma (1) |
| Oil (vehicle) | Mammary gland | Adenofibroma (4) |
| | | Fibroadenoma (7) |
| | | Fibroma (3) |
| | | Adenocarcinoma (1) |
| | Uterus | Leiomyofibrosarcoma (1) |
| | Kidney | Malignant nephroma (1) |
| | Pituitary | Adenocarcinoma (1) |
| | Adrenal gland | Phaeochromocytoma (1) |
| | Lymphatic tissue | Malignant lymphoma (1) |
| Untreated | Mammary gland | Adenofibroma (1) |
| | | Fibroadenoma (5) |
| | | Fibroma (3) |
| | | Adenocarcinoma (1) |
| | Uterus | Malignant mesenchymoma (1) |
| | | Leiomyofibrosarcoma (1) |
| | | Fibroadenoma (1) |
| | | Schwannoma (1) |
| | Abdomen | Adenocarcinoma (4) |
| | | Fibroma (1) |
| | Pituitary | Adenoma (1) |
| | Adrenal gland | Cortical adenocarcinoma (1) |
| | Islets of Langerhans | Islet-cell carcinoma (1) |
| | Lymphatic tissue | Malignant lymphoma (1) |

FIG. 7.—Incidences of animals with stomach tumours and reactive changes of the squamous epithelium of the stomach amongst the rats administered ethylene oxide.

FIG. 8.—Incidences of animals with stomach tumours and reactive changes of the squamous epithelium of the stomach amongst the rats administered 1,2-propylene oxide.

tration induces malignant tumours in the stomach of the rat. It is unlikely that these tumours could have been caused by the infection which appeared in our rats in the course of the experiment or by the agents employed in its treatment, despite the claim that chloramphenicol might possess carcinogenic activity (Schmähl, 1977; IARC, 1978), since the control animals in this experiment, as well as another 100 rats in another experiment did not develop any stomach tumours, although they were affected by the infection and treated in the same way.

$\beta$-Propiolactone produced a high incidence of stomach tumours in our experiment. The incidence of tumours was higher than that produced by ethylene oxide or by 1,2-propylene oxide. Furthermore, the tumours were generally larger than those induced by either of the 2 test compounds and were in all cases the primary cause of death.

Thus the carcinogenic effect of $\beta$-propiolactone in our experiment is more potent than that produced by ethylene oxide or propylene oxide. $\beta$-Propiolactone was investigated by Van Duuren *et al.* (1966) *via* the intragastric route and was also found to be a potent carcinogen. These authors, however, failed to obtain any positive results with the suspected carcinogens d,l-diepoxybutane and glycidaldehyde. It is likely that the regimen of treatment employed in our experiment (*viz.*, the twice weekly administration instead of once weekly and the greater number of animals) may have been important factors in enabling us to achieve a positive result. Furthermore, the use of a larger volume of solvent may have improved the absorption of the test substance into the epithelium of the stomach. The importance of the method of treatment in carcinogenicity studies of ethylene oxide by the oral route is demonstrated by the negative results obtained when rodent food fumigated by a high concentration of ethylene oxide was administered to rats for the whole of their life-time. The slow release of the compound from solid food under the latter conditions may have prevented a sufficient amount from penetrating the epithelium (Bär & Griepentrog, 1969).

In earlier experiments we were able to show that the carcinogenic activity of ethylene oxide and 1,2-propylene oxide differ slightly from each other upon s.c. application (Dunkelberg, 1979, 1981). However, considerable differences become evident upon intragastric administration, when ethylene oxide is shown to be more efficient than 1,2-propylene oxide. In the case of the low pH value of the empty stomach (pH ~1) the various acid-catalysed hydrolysis of the 2 compounds could considerably influence carcinogenicity. According to Ehrenberg & Hussain (1981) the estimated half-life of ethylene oxide at pH 1 and 37°C is about 3·5 min and that of 1,2-propylene oxide about 1 min. Ethylene oxide is therefore somewhat more stable than 1,2-propylene oxide under these conditions. Both compounds are converted relatively quickly in the acidic stomach juice. This is consistent with the findings that ethylene oxide and 1,2-propylene oxide induced tumours mainly in the forestomach and only rarely in the glandular stomach. In contrast to the forestomach the epithelium of the glandular stomach is apparently less exposed to attack by the 2 test compounds due to the protective influence of the formation of juice pepsin-hydrochloric acid. Because of the evidence of carcinogenic activity of ethylene oxide new aspects of the hygienic-toxicological evaluation of this compound in relation to its various areas of application such as the chemical, pharmaceutical and food industries, as well as the medical sphere, must be considered. The metabolic formation of 1,2-alkene epoxides from corresponding alkenes must, however, also be taken into account. It was established by Ehrenberg *et al.* (1977) that male CBA mice which were exposed to air contaminated with [$^{14}$C]-labelled ethene were able to metabolize this olefine to ethylene oxide.

The author would like to thank Prof. Dr U. Mohr, of the Medizinische Hochschule Hannover, for the histological findings, the Deutsche Forschungsgemeinschaft, Bonn, for the financial support of this study and Miss B. Haacker for technical assistance.

### REFERENCES

Bär, F. & Griepentrog, F. (1969) Langzeitfutterungsuersuch an Ratten mit Athylenoxide-begastum Futter. *Bundesgesundhbl*, **12**, 106.

Druckrey, H., Preussmann, R., Ivankovic, S., So, B. T., Schmidt, C. H. & Bücheler, J. (1966) Zur Erzeugung subcutaner Sarkome an Ratten Carcinogene Wirkung von Hydrazodicarbonsäure-bis-(methyl-nitrosamid), N-Nitroso-N-n-butyl-harnstoff, N-Methyl-N-nitroso-nitroguanidin und N-Nitroso-imidazolidon. *Z. Krebsforsch.*, **68**, 87.

Dunkelberg, H. (1979) On the oncogenic activity of ethylene oxide and propylene oxide in mice. *Br. J. Cancer*, **39**, 588.

Dunkelberg, H. (1981) Carcinogenic activity of ethylene oxide in comparison with 1,2-propylene oxide after subcutaneous administration in mice. *Zbl. Bakt. Hyg.* (I. Abt. Orig. B), **174**, 383.

Ehrenberg, L., Hiesche, K. D., Osterman-Golkar, S. & Wennberg, J. (1974) Evaluation of genetic risks of alkylating agents: tissue doses in the mouse from air contaminated with ethylene oxide. *Mutat. Res.*, **24**, 83.

Ehrenberg, L., Osterman-Golkar, S., Segerback, D., Svensson, K. & Calleman, C. J. (1977) Evaluation of genetic risk of alkylating agents. III. Alkylation of haemoglobin after metabolic conversion of ethene to ethene oxide in vivo. *Mutat. Res.*, **45**, 175.

Ehrenberg, L. & Hussain, S. (1981) Genetic toxicity of some important epoxides. *Mutat. Res.*, **86**, 1.

Fraenkel-Conrat, H. (1961) Chemical modification of viral ribonucleic acid. I. Alkylating agents. *Biochim. Biophys. Acta*, **49**, 169.

Glaser, Z. R. (1979) Ethylene oxide toxicology review and field study results of hospital use. *J. Environ. Pathol. Toxicol.*, **2**, 173.

Hogstedt, C., Malmqvist, N. & Wadmann, B. (1979*a*) Leukemia in workers exposed to ethylene oxide. *J. Am. Med. Ass.*, **241**, 1132.

Hogstedt, C., Rohlen, O., Berndtsson, B. S., Axelson, O. & Ehrenberg, L. (1979*b*) A cohort study of mortality and cancer incidence in ethylene oxide production workers. *Br. J. Ind. Med.*, **36**, 276.

Hogsted, C., Rohlen, O., Berndtsson, B. S., Axelsen, O. & Ehrenberg, L. (1978) Kohortstudie av dödsorsaker hos anställda i etylenoxidframstälning. *Läkertidningen*, **75**, 3285.

International Agency for Research on Cancer (1978) Chemicals with sufficient evidence of carcinogenicity in experimental animals-IARC Monographs Volumes 1–17. *Internal Technical Report No. 78/003*, Lyon: IARC.

La Borde, J. B. & Kimmel, C. A. (1981) The teratogenicity of ethylene oxide administered intravenously to mice. *Toxicol. Appl. Pharmacol.*, **56**, 16.

LAWLEY, P. D. & JARMAN, M. (1972) Alkylation by propylene oxide of deoxyribonucleic acid, adenine, guanosine and deoxyguanylic acid, *Biochem. J.*, **126**, 893.

LAWLEY, P. D. & WALLICK, C. A. (1957) The action of alkylating agents on deoxyribonucleic acid and guanylic acid. *Chem. Ind.*, 633.

REYNIERS, J. A. SACKSTEDER, M. R. & ASHBURN, L. L. (1964) Multiple tumors in female germfree inbred albino mice exposed to bedding treated with ethylene oxide. *J. Natl Cancer Inst.*, **32**, 1045.

SCHMÄHL, D. (1977) Toxikologie in der krebsforschung. II Kanzerogene Ärzneimittel. *Dtsch. Med. Wschr.*, **102**, 1047.

SCHÖNFELDT, N. (1976) *Grenzflächenaktive Äthylen-oxid-Addukte, ihre Herstellung, Eigenschaften, Anwendung und Analyse.* Stuttgart: Wiss Verlags-gesellschaft. p. 7.

VAN DUUREN, B. L., LANGSETH, L., ORRIS, L., TEEBOR, G., NELSON, N. & KUSCHNER, M. (1966) Carcinogenicity of epoxides, lactones and peroxy compounds. IV. Tumor response in epithelial and connective tissue in mice and rats. *J. Natl Cancer Inst.*, **37**, 825.

WALPOLE, A. L. (1958) Carcinogenic action of alkylating agents. *Ann. N.Y. Acad. Sci.*, **68**, 750.

WINDMUELLER, H. G. & KAPLAN, N. O. (1961) The preparation and properties of N-hydroxyethyl derivatives of adenosine, adenosine triphosphate and nicotinamide adenine dinucleotide. *J. Biol. Chem.*, **236**, 2716.

WOLMAN, S. R. (1979) Mutational consequences of exposure to ethylene oxide. *J. Environ. Pathol., Tocixol.*, **2**, 1289.


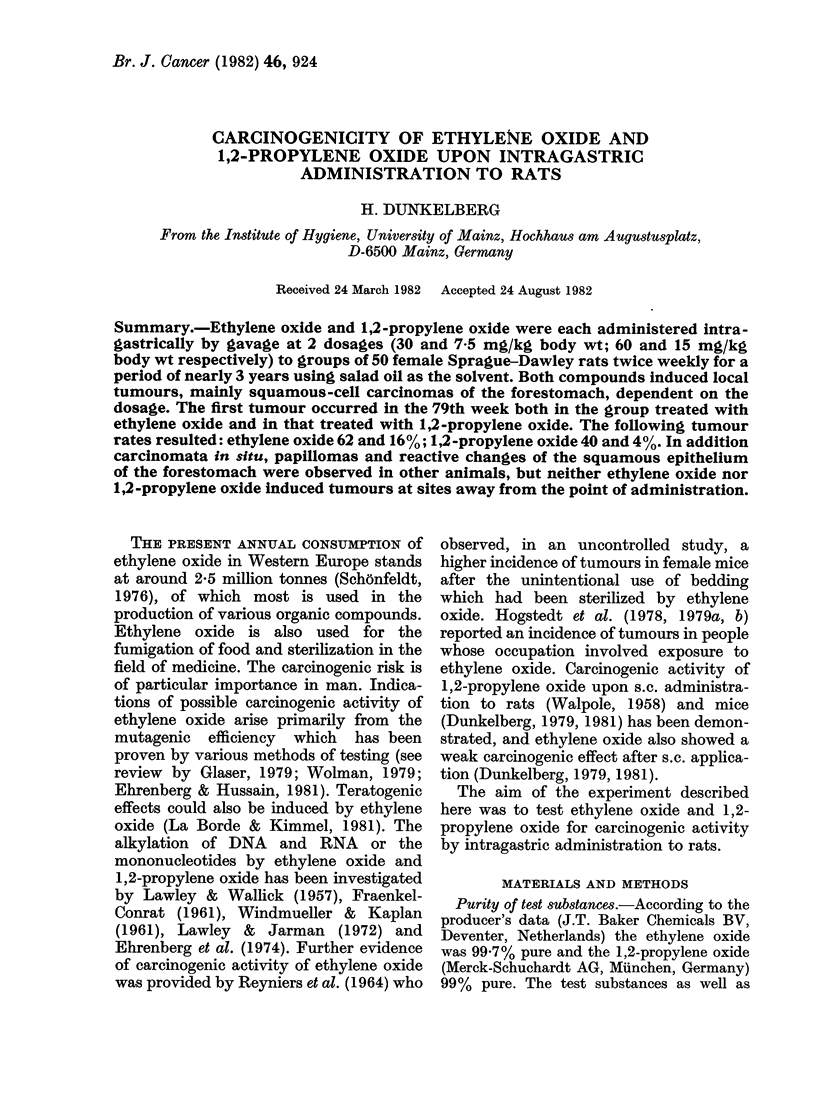

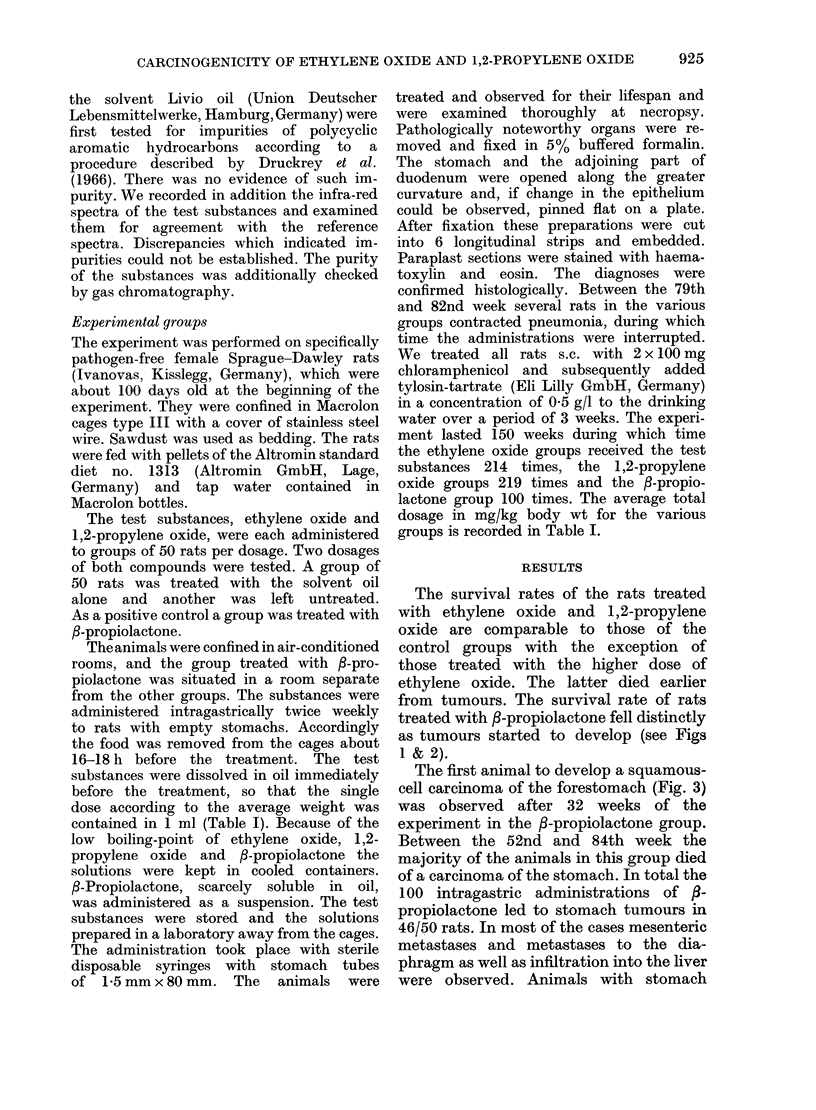

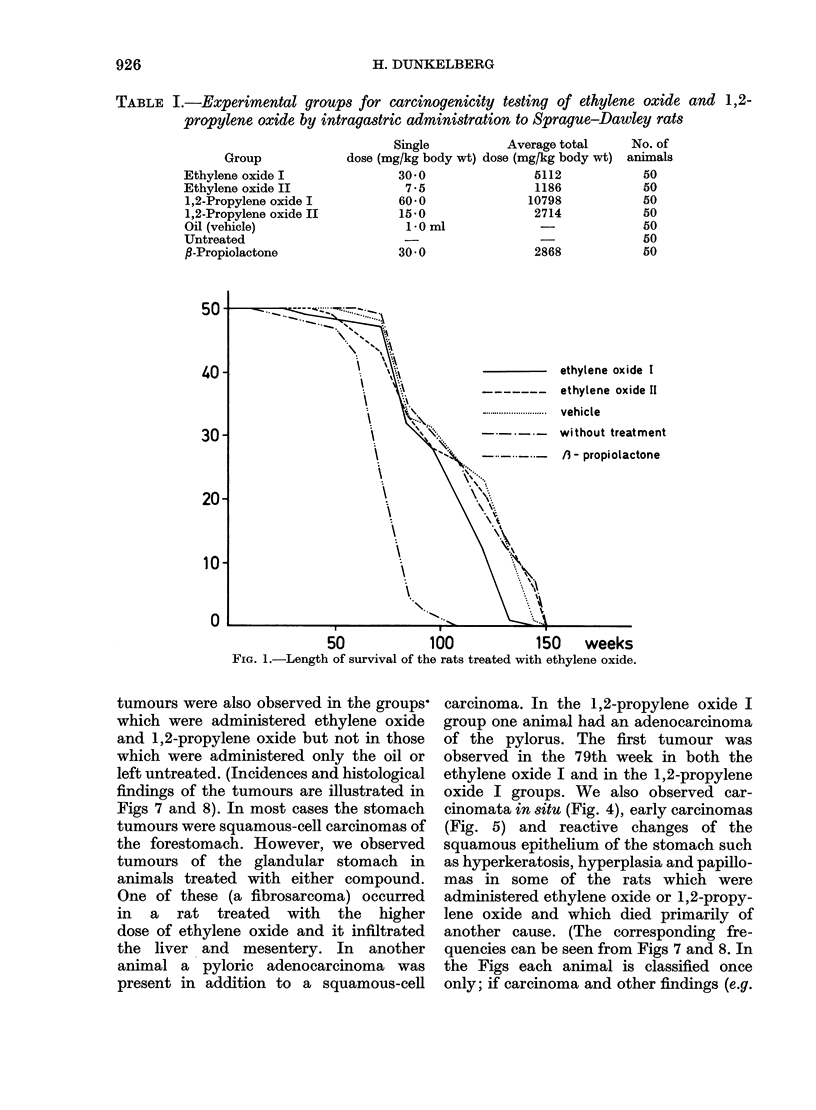

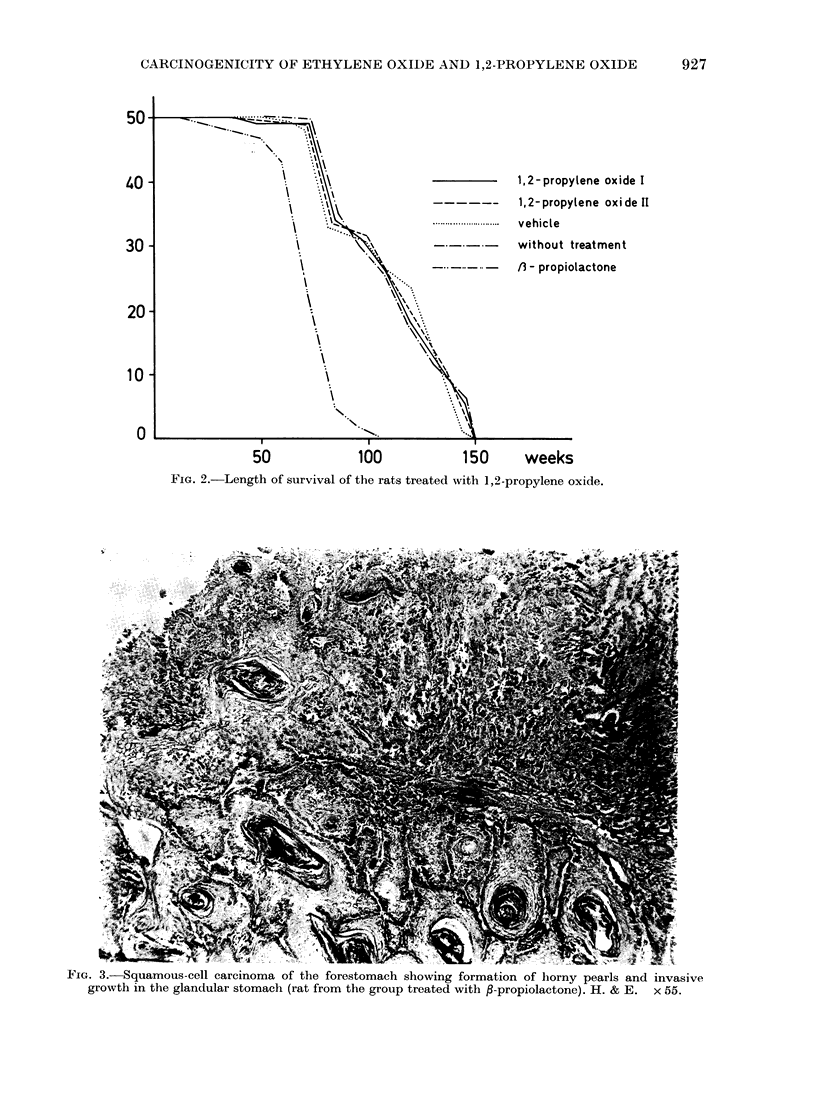

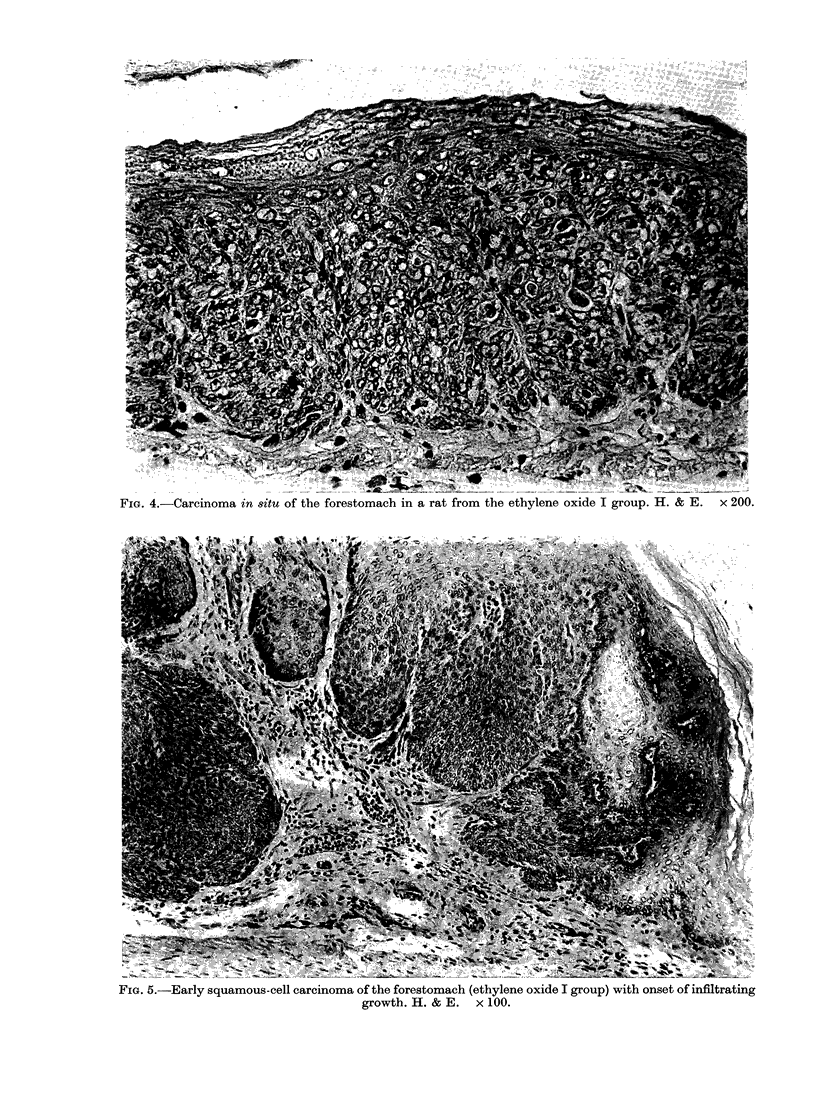

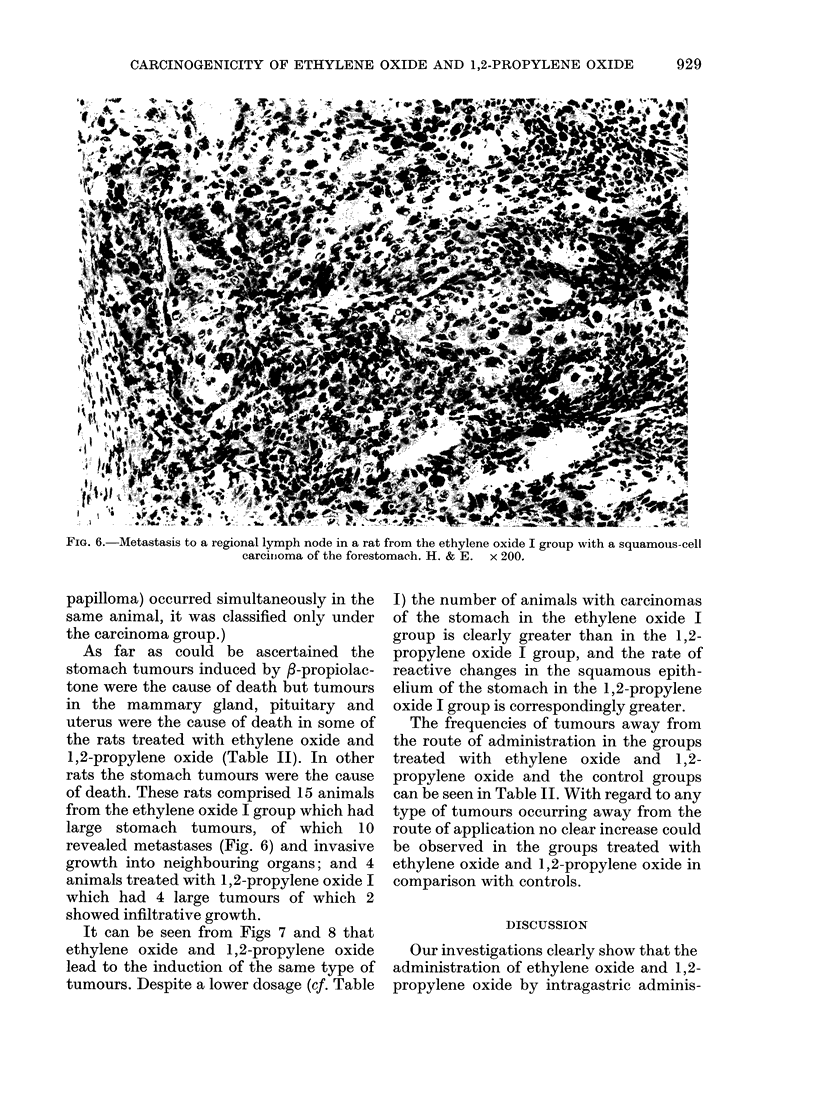

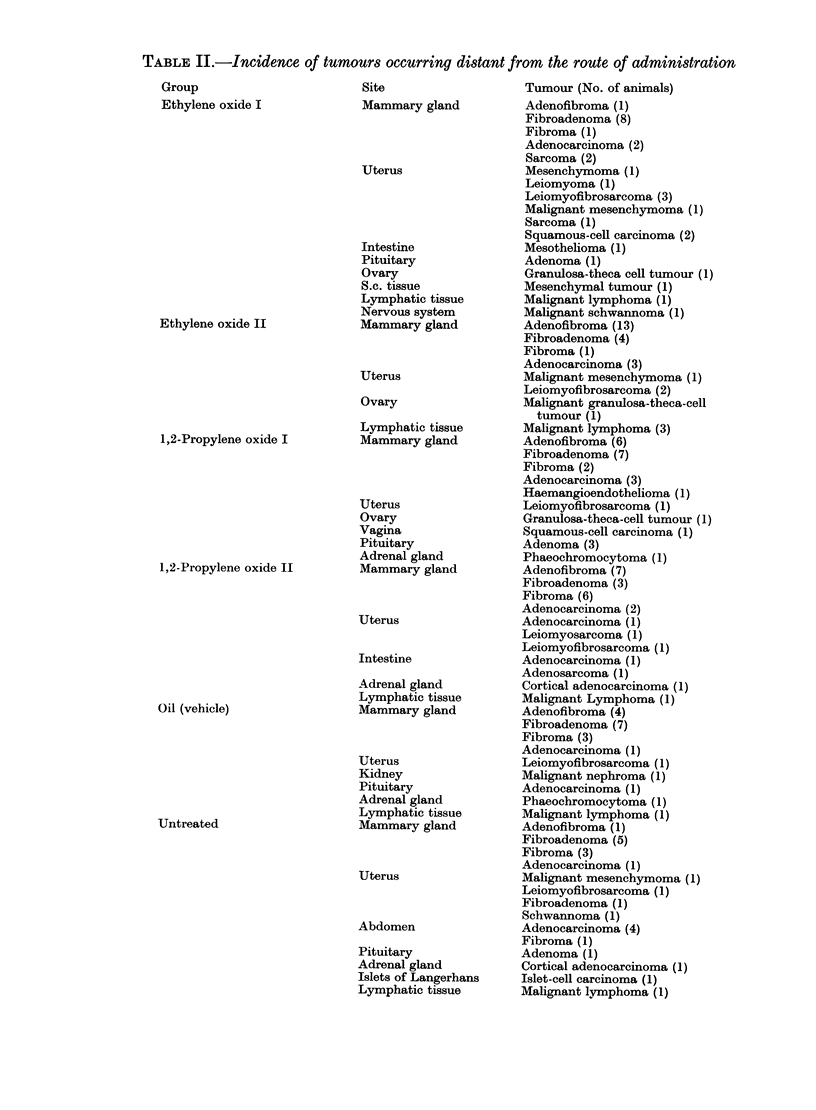

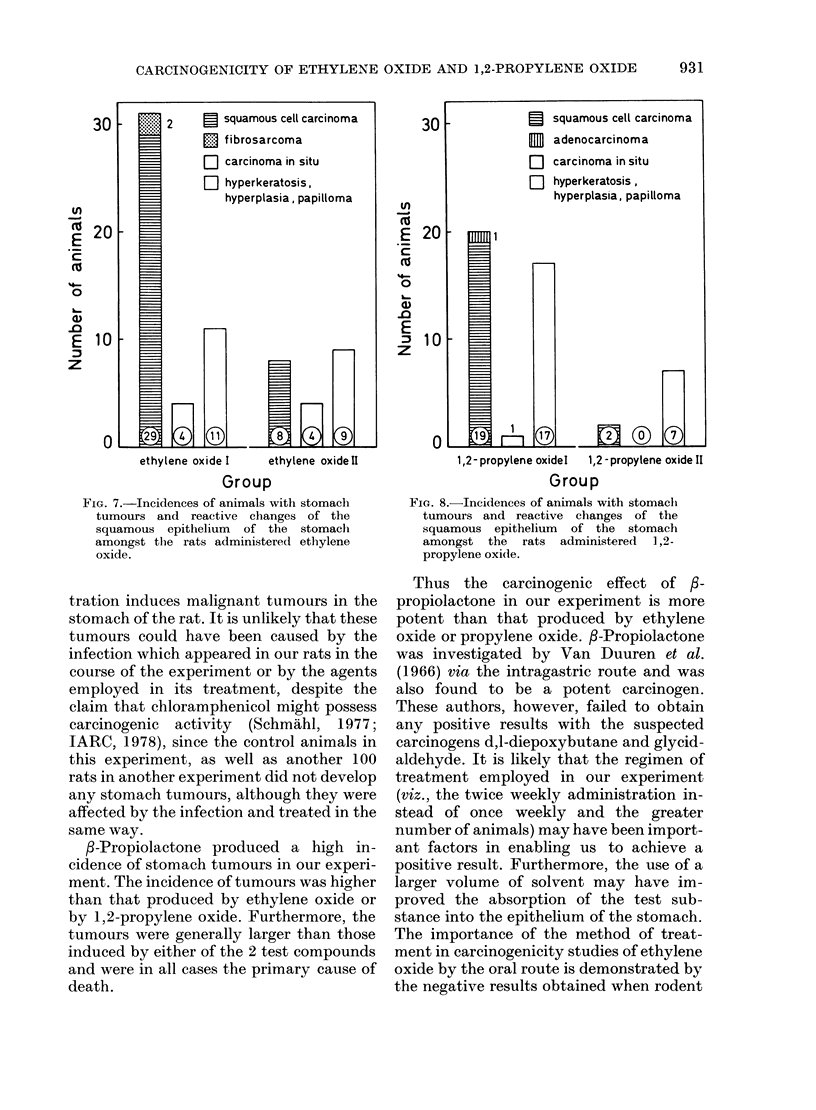

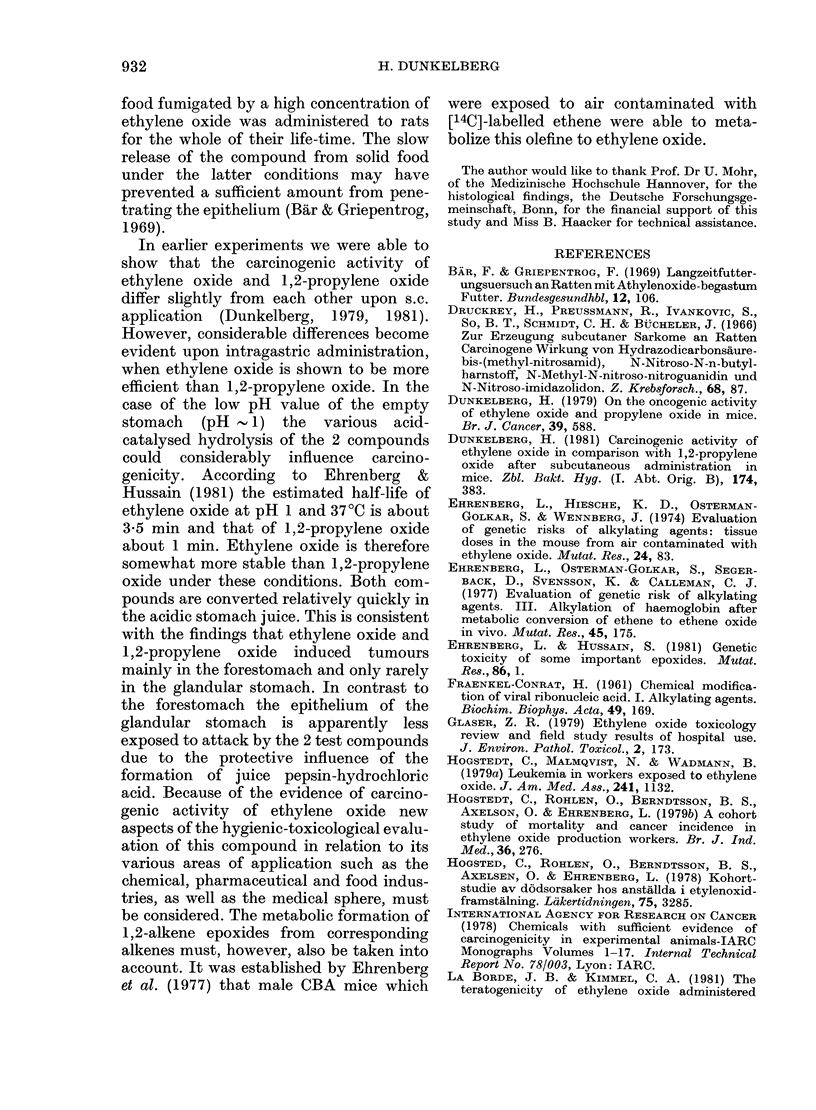

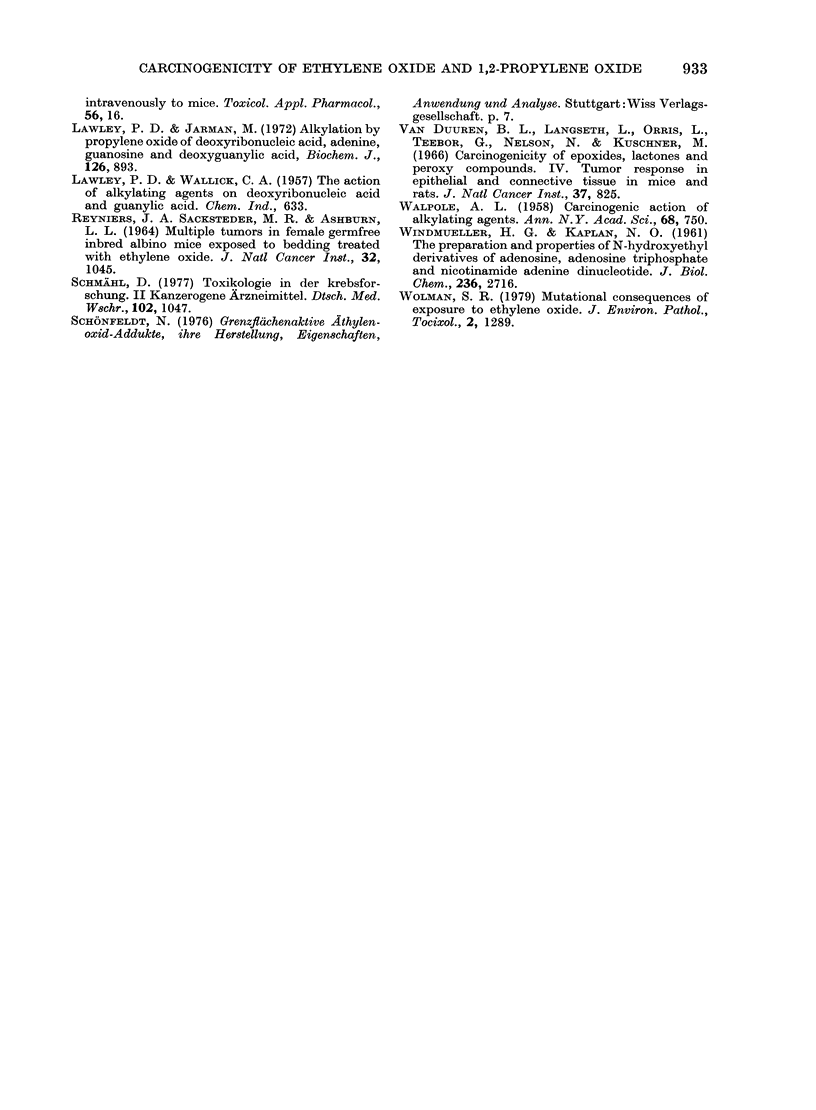


## References

[OCR_00748] Druckrey H., Preussmann R., Ivankovic S., So B. T., Schmidt C. H., Bücheler J. (1966). Zur Erzeugung subcutaner Sarkome an Ratten. Carcinogene Wirkung von Hydrazodicarbonsäure-bis-(methyl-nitrosamid), N-Nitroso-N-n-butyl-harnstoff, N-Methyl-N-nitroso-nitroguanidin und N-Nitroso-imidazolidon.. Z Krebsforsch.

[OCR_00762] Dunkelberg H. (1981). Kanzerogene Aktivität von Ethylenoxid und seinen Reaktionsprodukten 2-Chlorehtnaol, 2-Bromethanol, Ethylenglykol und Diethylenglykol. I. Kanzerogenität von Ethylenoxid im Vergleich zu 1,2-Propylenoxid bei subkutaner Applikation an Mäusen.. Zentralbl Bakteriol Mikrobiol Hyg B.

[OCR_00757] Dunkelberg H. (1979). On the oncogenic activity of ethylene oxide and propylene oxide in mice.. Br J Cancer.

[OCR_00771] Ehrenberg L., Hiesche K. D., Osterman-Golkar S., Wenneberg I. (1974). Evaluation of genetic risks of alkylating agents: tissue doses in the mouse from air contaminated with ethylene oxide.. Mutat Res.

[OCR_00785] Ehrenberg L., Hussain S. (1981). Genetic toxicity of some important epoxides.. Mutat Res.

[OCR_00778] Ehrenberg L., Osterman-Golkar S., Segerbäck D., Svensson K., Calleman C. J. (1977). Evaluation of genetic risks of alkylating agents. III. Alkylation of haemoglobin after metabolic conversion of ethene to ethene oxide in vivo.. Mutat Res.

[OCR_00790] FRAENKEL-CONRAT H. (1961). Chemical modification of viral ribonucleic acid. I. Alkylating agents.. Biochim Biophys Acta.

[OCR_00795] Glaser Z. R. (1979). Ethylene oxide: toxicology review and field study results of hospital use.. J Environ Pathol Toxicol.

[OCR_00800] Hogstedt C., Malmqvist N., Wadman B. (1979). Leukemia in workers exposed to ethylene oxide.. JAMA.

[OCR_00805] Hogstedt C., Rohlén O., Berndtsson B. S., Axelson O., Ehrenberg L. (1979). A cohort study of mortality and cancer incidence in ethylene oxide production workers.. Br J Ind Med.

[OCR_00825] LaBorde J. B., Kimmel C. A. (1980). The teratogenicity of ethylene oxide administered intravenously to mice.. Toxicol Appl Pharmacol.

[OCR_00834] Lawley P. D., Jarman M. (1972). Alkylation by propylene oxide of deoxyribonucleic acid, adenine, guanosine and deoxyguanylic acid.. Biochem J.

[OCR_00845] REYNIERS J. A., SACKSTEDER M. R., ASHBURN L. L. (1964). MULTIPLE TUMORS IN FEMALE GERMFREE INBRED ALBINO MICE EXPOSED TO BEDDING TREATED WITH ETHYLENE OXIDE.. J Natl Cancer Inst.

[OCR_00864] Van Duuren B. L., Langseth L., Orris L., Teebor G., Nelson N., Kuschner M. (1966). Carcinogenicity of epoxides, lactones, and peroxy compounds. IV. Tumor response in epithelial and connective tissue in mice and rats.. J Natl Cancer Inst.

[OCR_00872] WALPOLE A. L. (1958). Carcinogenic action of alkylating agents.. Ann N Y Acad Sci.

[OCR_00875] WINDMUELLER H. G., KAPLAN N. O. (1961). The preparation and properties of N-hydroxyethyl derivatives of adenosine, adenosine triphosphate, and nicotinamide adenine dinucleotide.. J Biol Chem.

[OCR_00882] Wolman S. R. (1979). Mutational consequences of exposure to ethylene oxide.. J Environ Pathol Toxicol.

